# Changes in Lysine Methylation Contribute to the Cytotoxicity of Curcumin in Colon Cancer Cells

**DOI:** 10.3390/molecules30020335

**Published:** 2025-01-16

**Authors:** Roberta Santarelli, Paola Currà, Michele Di Crosta, Roberta Gonnella, Maria Saveria Gilardini Montani, Mara Cirone

**Affiliations:** Department of Experimental Medicine, Sapienza University of Rome, 00161 Rome, Italy; roberta.santarelli@uniroma1.it (R.S.); curra.1999148@studenti.uniroma1.it (P.C.); michele.dicrosta@uniroma1.it (M.D.C.); roberta.gonnella@uniroma1.it (R.G.)

**Keywords:** colon cancer, curcumin, acetylation, methylation, mutp53, wtp53, methyltransferase, epigenetic

## Abstract

Epigenetic abnormalities play a critical role in colon carcinogenesis, making them a promising target for therapeutic interventions. In this study, we demonstrated that curcumin reduces colon cancer cell survival and that a decrease in lysine methylation was involved in such an effect. This correlated with the downregulation of methyltransferases EZH2, MLL1, and G9a, in both wild-type p53 (wtp53) HCT116 cells and mutant p53 (mutp53) SW480 cells, as well as SET7/9 specifically in wtp53 HCT116 cells. The effects induced by curcumin were more pronounced in wtp53 cells, where it induced a stronger apoptosis and ferroptosis. Interestingly, curcumin also reduced mutp53 expression, suggesting that it could enhance the efficacy of other therapies, particularly in overcoming drug resistance mechanisms associated with mutp53. For instance, in this study, we show that curcumin sensitized SW480 cells to SET7/9 inhibition by sinefungin, further supporting its potential as a combinatorial therapeutic agent. However, although to a lesser extent, curcumin also impaired cell survival in HCT 116 p53 null cells, suggesting that other molecular pathways or factors, beyond p53, may be involved in curcumin-induced cytotoxicity.

## 1. Introduction

Curcumin is a bioactive compound derived from Curcuma longa, an herbaceous plant in the ginger family [1]. As for other natural compounds [2], curcumin has been identified as a promising therapeutic agent for a range of diseases, including neurodegenerative disorders and cancer. One of curcumin’s key beneficial effects is its ability to reduce chronic inflammation, which contributes to these pathological conditions [3,4]. Curcumin has been shown to induce apoptosis caused by endoplasmic reticulum (ER) stress in cancer cells [5] and target multiple pathways critical to both inflammation and carcinogenesis, such as STAT3 and NF-κB [6]. This is important as the concomitant inhibition of these pathways may help to prevent the activation of feedback resistance mechanisms, thereby enhancing therapeutic efficacy.

Curcumin is one of the more effective epigenetic modulators of natural origin and its therapeutic effects involve epigenetic changes [7]. These include the upregulation of enzymes responsible for DNA methylation such as DNMT1 and DNMT3 [8], those mediating lysine acetylation of histones and non-histone proteins such as histone acetyltransferases (HATs) and histone deacetylases (HDACs) [9], and those involved in lysine methylation [10].

Epigenetic alterations alongside genetic mutations contribute to carcinogenesis but, differently from genetic abnormalities, can be reverted by appropriate treatments. For this reason, there is a growing interest in this field, for the prevention and treatment of cancer. Epigenetic abnormalities in cancer include aberrant histone acetylation and methylation, where the first induces an activation of gene transcription while methylation can either activate or repress transcription, depending on the sites of methylation and on how many methyl groups are added [11]. It has been reported that, as a consequence of these epigenetic changes, the expression level of oncogenes and oncosuppressors may be altered, leading to the activation of the former and inactivation of the latter. Among the molecules playing a key role in carcinogenesis there is wild-type p53 (wtp53), the most important oncosuppressor which is often inactivated in cancer by mutations and/or by epigenetic modifications. Mutations can transform p53 into an oncogene that drives cancer instead of counteracting it [12], and thus the search for strategies aimed at reducing mutant p53 (mutp53) expression level and reactivate wtp53 is warranted. Among these, p53 acetylation or methylation may be exploited to regulate the function of wtp53 and mutp53 [13]. Histone and non-histone methylation are mediated by a variety of methyltransferases that, in some cases, share the targeted residues on histones. These enzymes include EZH2, G9a, and MLL1 that can methylate wtp53 to inhibit it and others such as SET7/9 that instead contribute to wtp53 activation [14]. However, methylation is a dynamic process and enzymes mediating de-methylation, such as KDM1, may counteract methylation mediated by SET7/9 and SET8 [12]. Regarding the impact of methylation on mutp53, no clear evidence is available in the current literature, meaning that deeper investigations are required to elucidate how it may regulate this protein.

Targeting epigenetic abnormalities contributing to carcinogenesis by using natural products instead of synthetic drugs may offer the advantage of minimizing the collateral effects while maintaining the efficacy of the treatments. Based on this background, and knowing that curcumin is an epigenetic modulator, in this study we evaluated whether this compound could alter lysine acetylation and methylation in colon cancer cell lines that carry wtp53 or mutp53, HCT116 and SW480, respectively. Since we found that methylation was modified more strongly than acetylation by curcumin, we focused on the expression of some methyltransferases and correlated their expression and activity on epigenetic markers as H3K27, H3K4, and H3K9 and the cytotoxic effect of curcumin against these tumor cells. We chose colon cancer to perform our experiments, as curcumin may be introduced through food and directly interact with colon epithelial cells and since epigenetic abnormalities play a key role in driving colon carcinogenesis.

## 2. Results

### 2.1. Curcumin Induces Colon Cancer Cell Apoptosis and Ferroptosis, Particularly in Cells Carrying wtp53

**Figure 1:**
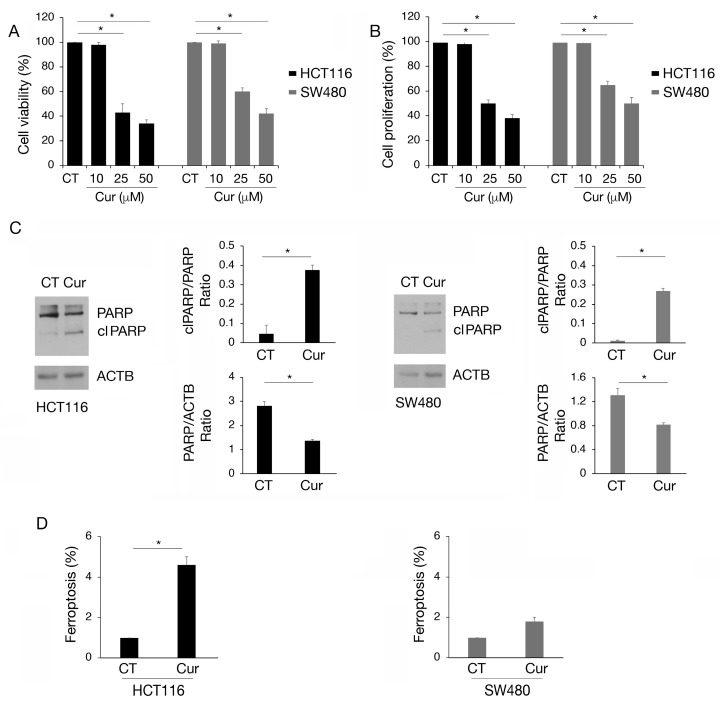
Apoptosis and ferroptosis are induced by curcumin in colon cancer cells, mainly in HCT116 carrying wtp53. (**A**) Trypan blue assay on HCT116 and SW480 cell lines cultured in presence of Cur 10, 25, and 50 μM for 24 h. Untreated cells were used as control (CT). (**B**) MTT assay to evaluate cell proliferation of HCT116 and SW480 cultured in presence of Cur 10, 25, and 50 μM for 24 h. The histograms represent the mean plus SD of three different experiments. (**C**) Western blot analysis to detect total PARP and cleaved (cl) PARP in HCT116 and SW480 cell lines cultured in presence of Cur 25 μM for 24 h. The histograms represent the mean plus SD of the densitometric analysis of the ratio of cl PARP/PARP and PARP/ACTB of three different experiments. (**D**) Detection and quantification of 4-hydroxynonenal (4-HNE) protein adducts in HCT116 and SW480 cells untreated or treated with Cur 25 μM for 24 h as a marker of ferroptosis. * indicates *p*-value < 0.05.

HCT116 and SW480 colon cancer cell lines, carrying wtp53 and mutp53, respectively, were treated with increasing doses of curcumin (Cur 10, 25, and 50 µM) for 24 h and cell survival was investigated by trypan blue assay. As shown in Figure 1A, curcumin exerted a stronger cytotoxic effect against wtp53 HCT116 compared to SW480 cells and these results were confirmed by performing an MTT assay, in the presence or in the absence of curcumin (Figure 1B). Moreover, curcumin was able to reduce cell survival, although to a lesser extent, in HCT116 p53 null cells (Figure S1A), while it slightly affected that of primary colonic epithelial cells (Figure S1B). This suggests that p53 was not the only molecule affected by curcumin in the first case and that its use is safe in normal cells. We chose the 25 µM dose of curcumin for subsequent experiments, as it induced 50% cell death in HCT116 and approximately 30% in SW480 cells, allowing us to better explore the differences between these cell types. Searching for the type of cell death, we found that curcumin induced PARP cleavage (clPARP) (Figure 1C), suggesting the occurrence of apoptosis in both cell lines, although this effect was more evident in wtp53 cells. We observed that curcumin also induced ferroptosis in HCT116 and slightly in SW480, as evidenced by the increase in 4-hydroxy-2-nonenal (4-HNE), a cytotoxic lipid peroxidation marker (Figure 1D). Ferroptosis is a type of cell death driven by iron-dependent phospholipid peroxidation which has been recently reported to be induced by curcumin in colon cancer [15,16]. Altogether these findings suggest that curcumin was able to impair colon cancer cell survival by inducing different types of cell death and that these effects were more evident in HCT116 cells compared to SW480 cells.

### 2.2. Curcumin Increases the Expression of wtp53 While Reducing That of mutp53 in Colon Cancer Cells

**Figure 2:**
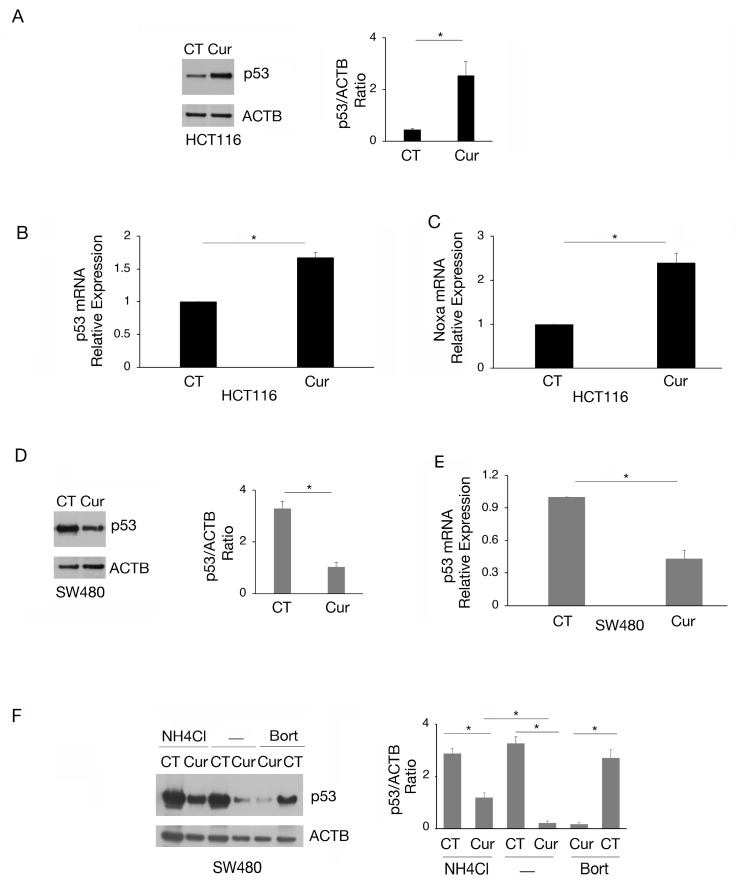
The expression of wtp53 is increased while that of mutp53 is reduced by curcumin in colon cancer cells. (**A**) wtp53 level assayed by Western blot analysis in HCT116 cell line cultured in presence of Cur 25 μM for 24 h. The histograms represent the mean plus SD of the densitometric analysis of the ratio of p53/ACTB of three different experiments. Untreated cells were used as control (CT). (**B**) RT-qPCR to analyze p53 expression in HCT116 cell line cultured in presence of Cur 25 μM for 24 h. (**C**) Noxa mRNA of HCT116 cells cultured in presence of Cur 25 μM for 24 h was quantified by RT-qPCR. (**D**) Level of mutp53 evaluated by Western blot analysis in SW480 cell line cultured in presence of Cur 25 μM for 24 h. The histograms represent the mean plus SD of the densitometric analysis of the ratio of p53/ACTB of three different experiments. (**E**) p53 mRNA expression of SW480 cells cultured in the presence of Cur 25 μM for 24 h was assessed by RT-qPCR. (**F**) Western blot analysis showing the level of mutp53 in SW480 cell line cultured in presence of Cur 25 μM for 24 h and incubated with NH_4_Cl 40 μM or bortezomib (20 nM), during the last 6 h, to inhibit phagosome–lysosome fusion or proteasomes. The histograms represent the mean plus SD of the densitometric analysis of the ratio of p53/ACTB of three different experiments. For all the RT-qPCRs in the figure, beta-actin was used as the reference gene and one representative experiment is shown and histograms represent the mean plus SD of three different experiments. * indicates *p*-value < 0.05.

According to the reduction in cell survival, we then found that curcumin induced wtp53 accumulation in HCT116 cells (Figure 2A), increased its mRNA expression (Figure 2B), and induced its activation, as evidenced by the upregulation of the proapoptotic target Noxa (Figure 2C). Interestingly, in SW480 cells, we found that curcumin downregulated the expression level of mutp53 (Figure 2D) due to the reduction in mRNA expression (Figure 2E) and the induction of lysosomal degradation. Indeed, mutp53 expression level was partially rescued by the lysosomal inhibitor NH_4_Cl while not by bortezomib (Figure 2F). This suggests that a proteasomal route was not involved in such an effect. The modifications of wt- and mutp53 expression level could contribute to the cytotoxic effect of curcumin observed in HCT116 and SW480 cells, knowing that either the activation of wtp53 or the reduction in mutp53, with pro-oncogenic functions, may promote cancer cell death.

### 2.3. Curcumin Reduces Lysine Methylation and Changes the Expression of mRNA Encoding for Several Methyltransferases in Colon Cancer Cells

**Figure 3:**
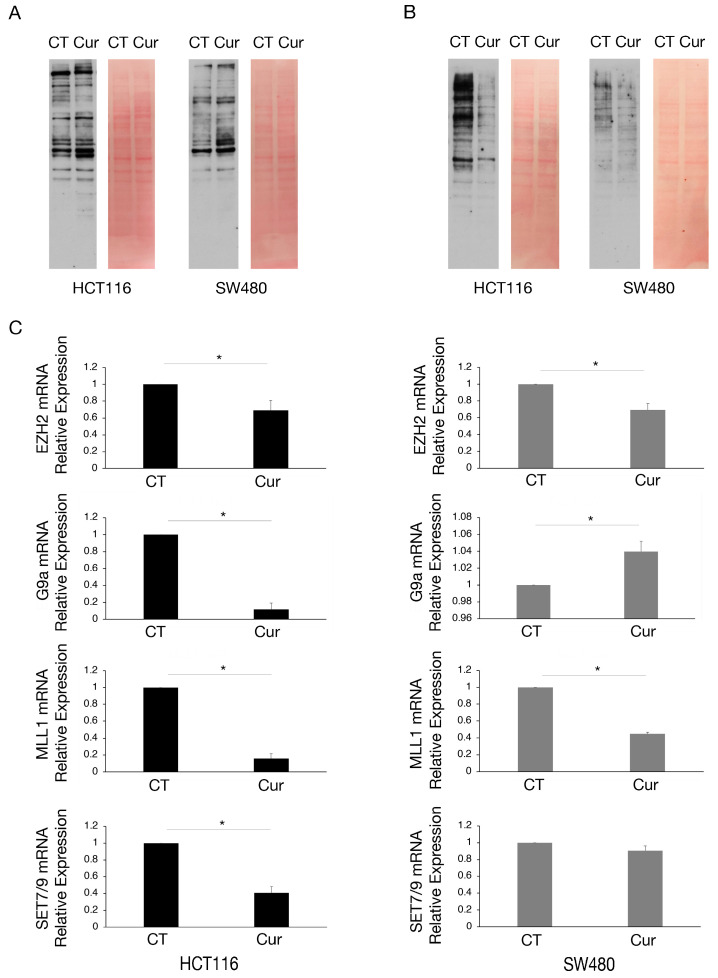
Lysine methylation and the expression of mRNA encoding for several methyltransferases are reduced by curcumin in colon cancer cells, mainly in HCT116 cells. Western blot analysis to assess (**A**) the lysine acetylation and (**B**) lysine methylation in HCT116 and SW480 cell lines cultured in presence of Cur 25 μM for 24 h. Untreated cells were used as control (CT). Membranes were stained with red Ponceau to evaluate the total protein amount of each sample. One representative experiment is shown. (**C**) EZH2, G9a, MLL1, and SET7/9 mRNA level of HCT116 and SW480 cells cultured in presence of Cur 25 μM for 24 h evaluated by RT-qPCR. For all the RT-qPCRs in the figure, beta-actin was used as the reference gene and one representative experiment is shown and histograms represent the mean plus SD of three different experiments. * indicates *p*-value < 0.05.

As curcumin is considered a natural epigenetic modulator, we explored if it could affect lysine methylation and/or acetylation in colon cancer cells. We found that lysine acetylation slightly increased (Figure 3A) while lysine methylation was strongly reduced by curcumin, an effect more evident in HCT116 cells that basally display a higher lysine methylation (Figure 3B).

We then investigated the expression of some methyltransferases involved in the survival of cancer cells, such as EZH2, G9a, MLL1, and SET7/9, in HCT116 and SW480 cells undergoing curcumin treatment. Therefore, we performed RT-qPCR and observed that EZH2 and MLL1 mRNAs were reduced by curcumin in both cell lines, while G9a and SET7/9 mRNAs were reduced only in HCT116 (Figure 3C), cells in which the demethylating effect of curcumin was more evident.

### 2.4. Methyltransferase Regulation by Curcumin Correlates with Histone Methylation

**Figure 4:**
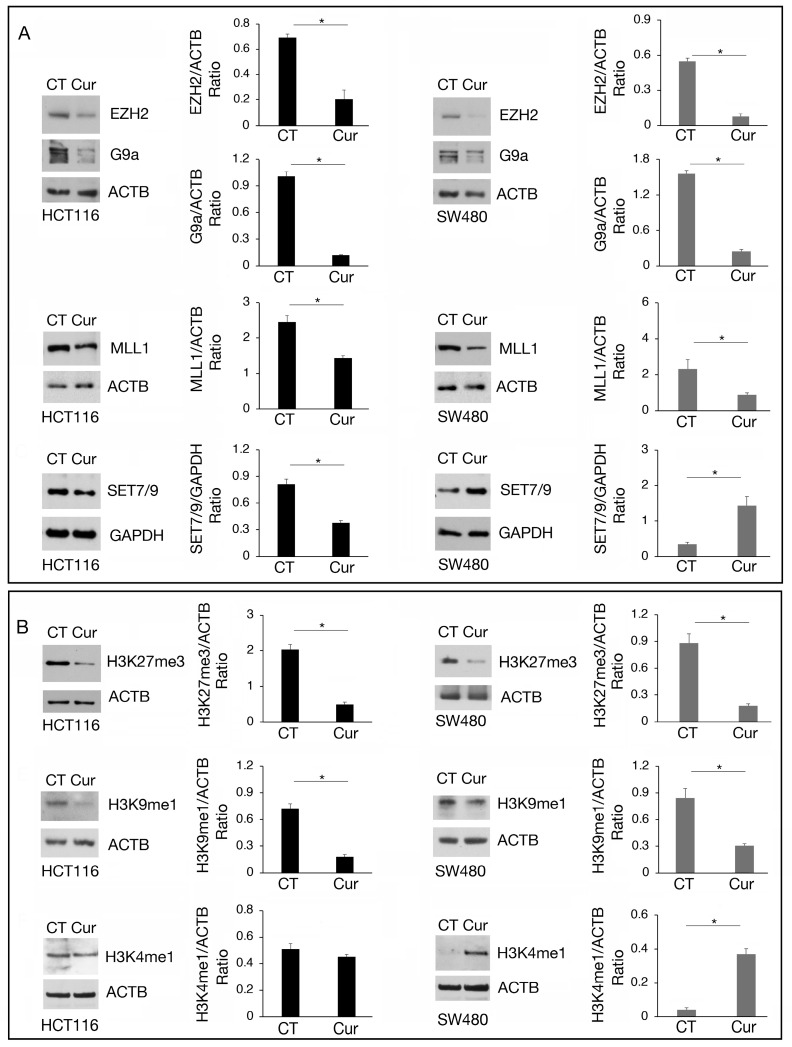
The modulation of EZH2, G9a, MLL1, and SET7/9 methyltransferases by curcumin correlates with the methylation of their target. Western blot analysis showing the level of (**A**) EZH2, G9a, MLL1, and SET7/9 and (**B**) H3K27me3, H3K9me1, and H3K4me1 in HCT116 and SW480 cell lines cultured in presence of Cur 25 μM for 24 h. Untreated cells were used as control (CT). The histograms represent the mean plus SD of the densitometric analysis of the ratio of EZH2/ACTB, G9a/ACTB, MLL1/ACTB, SET7/9/GAPDH, H3K27me3/ACTB, H3K9me1/ACTB, H3K4me1/ACTB of three different experiments. * indicates *p*-value < 0.05.

We then investigated the expression of the above-mentioned methyltransferases at the protein level. As shown in Figure 4A, EZH2, G9a, and MLL1 were downregulated by curcumin in both cell types while SET7/9 was downregulated in HCT116 and upregulated in SW480 cells, in correlation with the results obtained by RT-qPCR. Regarding G9a, we found that it was also downregulated at protein level in SW480 cells, suggesting a post-transcriptional regulation of the protein following curcumin treatment.

The methylation of histone residues targeted by these methyltransferases was then evaluated, keeping in mind that methylation of a specific target can be mediated by more than one methyltransferase and counterbalanced by demethylases. We found that the trimethylation of H3K27 (H3K27me3), the main target of EZH2, and of H3K9me1, the main target of G9a, was reduced in both HCT116 and SW480 cells treated by curcumin, while H3K4me1, target of SET7/9 and MLL1, was slightly affected in HCT116 and increased in SW480 (Figure 4B), in correlation with SET7/9 upregulation induced by curcumin in the latter cells.

### 2.5. EZH2, G9a, MLL1, and to a Lesser Extent SET7/9 Contribute to the Cytotoxic Effect of Curcumin, Particularly in wtp53 HCT116 Cells

**Figure 5:**
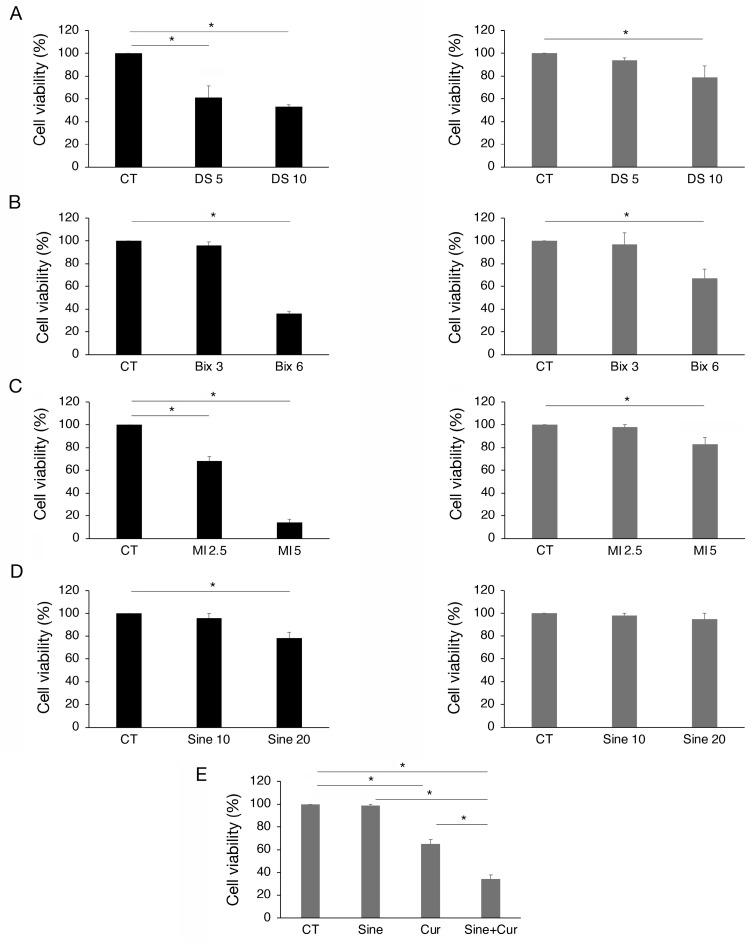
The inhibition of EZH2, MLL1, G9a, and SET7/9 reduces viability mainly in HCT116 cells. Trypan blue exclusion assay on HCT116 and SW480 cell lines cultured in presence of (**A**) DS-3201 (DS) EZH2 inhibitor, 5 and 10 μM; (**B**) Bix-01294 (Bix) G9a inhibitor, 3 and 6 μM; (**C**) MI-2 (MI) Menin inhibitor, 2.5 and 5 μM; (**D**) Sinefungin (Sine) SET7/9 inhibitor, 10 and 20 μM. (**E**) Trypan blue exclusion assay on SW480 cells cultured in presence of Sinefungin 10 μM and Cur 25 μM for 24 h. Untreated cells were used as control (CT). All the experiments were performed in triplicate and repeated three times. * indicates *p*-value < 0.05.

To investigate if the modifications induced by curcumin of EZH2, G9a, MLL1, and SET7/9 could contribute to its cytotoxic effect, we pharmacologically inhibited each methyltransferase. As shown in Figure 5A–C, the inhibition of EZH2, G9a, and MLL1 by Valemetostat (DS), BIX01294 (Bix), and menin inhibitor (MI-2), respectively, used at two different concentrations, reduced cell survival of HCT116 and slightly that of SW480 cells. Conversely, SET7/9 inhibition by sinefungin (Sine) was less involved in the survival of both cell lines (Figure 5D). However, as curcumin upregulated SET7/9 in SW480, we explored if its inhibition during curcumin treatment could potentiate the cytotoxic effect of this compound. We found that this was the case, suggesting that the upregulation of SET7/9 by curcumin was a resistance mechanism to its cytotoxic effect in SW480 cells (Figure 5E). Altogether these results suggest that the inhibition of EZH2, G9a, and MLL1 contributed to the cytotoxic effect of curcumin, particularly in wtp53-carrying cells, and that the upregulation of SET7/9 is involved in the resistance of the SW480 cell line to this compound.

## 3. Discussion

Histone methylation/demethylation regulates key biological processes such as gene transcription, nucleosomal positioning, and DNA repair. Dysregulating methylation may increase the risk of cancer as previously reported [17]. Colon cancer, one of the leading causes of cancer-related mortality, frequently exhibits abnormalities in methylation patterns [18]. Indeed, both mutations and epigenetic alterations are required in the different steps leading to malignant transformation of colon cells. Moreover, it has been reported that epigenetic alterations are involved in resistance to therapy and, consequently, a drug able to act on epigenetic modulators could help to overcome it [19].

Among anti-cancer agents, curcumin has garnered attention for its ability to modulate epigenetic processes in cancer, being able to influence both DNA and histone methylation besides its anti-inflammatory and anti-oxidant properties [20]. Curcumin and its derivatives have been shown to inhibit methyltransferase activity and downregulate histone methylation markers such as H3K4, H3K9, and H3K27 in a model of acute myeloid leukemia (AML) [10]. Histones epigenetic modifications such as acetylation and methylation are responsible for altered gene expression of oncogenes and tumor-suppressor genes underlying cancer development.

In the present study, we demonstrate that curcumin changes the methylation pattern and the expression of several methyltransferases in colon cancer cells carrying wtp53 and mutp53. Methyltransferases inducing repressive markers, such as EZH2 and G9a, and those inducing activation-associated markers like MLL1 and SET7/9, were affected. Specifically, G9a was found to be downregulated at the transcriptional level in wtp53 HCT116 cells, while in mutp53 SW480 cells the downregulation occurred at the post-transcriptional level.

Furthermore, curcumin exhibited a greater cytotoxicity that correlated with its stronger demethylating effects in HCT116 cells, which were characterized by higher basal levels of lysine methylation and greater sensitivity to the inhibition of EZH2, MLL1, and G9a. It has been previously demonstrated the inhibition of EZH2, MLL1, and G9a may be a strategy to impair the survival of various tumor types, underscoring their significance as critical therapeutic targets in cancer [21,22,23].

Besides the lower sensitivity to EZH2, MLL1, and G9a, here we found that the upregulation of the methyltransferase SET7/9 in mutp53 cells was also a resistance mechanism to curcumin, as indeed its inhibitor sinefungin potentiated the cytotoxicity of curcumin treatment. Therefore, the presence of mutp53 appears to act as a resistance mechanism against curcumin as well as the reduction in these methyltransferases, as previously observed with other anti-cancer treatments [24]. Notably, curcumin was able to reduce mutp53 expression at both transcriptional and post-transcriptional levels in colon cancer cells, as lysosomes but not proteasomes partially rescued mutp53 expression level. Given the pro-oncogenic role that mutp53 can acquire [25], this mechanism may contribute to the impairment of cell survival, particularly in combination with other treatments. However, although to a lesser extent, curcumin reduced cell survival in HCT116 p53 null cells, which may suggest a broader effect of this natural compound and that other oncogenic molecules or oncosuppressors could be affected.

As a mechanism leading to cell death, curcumin induced PARP cleavage, suggesting apoptotic cell death, and also triggered ferroptosis, although both effects and particularly ferroptosis were more pronounced in wtp53 cells compared to mutp53 cells. Interestingly PARP cleavage and the consequent reduction in PARP could help to facilitate ferroptosis, thereby bridging ferroptosis and apoptosis in curcumin-treated cancer cells [26].

The different extents of ferroptosis observed in wt- and mutp53 cells may be due to the fact that, unlike wtp53, mutp53 is unable to repress function and can even upregulate the cystine/glutamate transporter SLC7A11, which increases the capacity of tumor cells to resist ferroptosis by increasing glutathione-mediated anti-oxidant defense [26].

In conclusion, in the present study, we show for the first time that curcumin alters the expression of histone methyltransferases in colon cancer cells and that these changes contribute to its cytotoxic effect, although the potential impact of histone acetylation and methylation changes on specific gene regulation remains to be explored. The cytotoxic effect of curcumin was lower against mutp53 cells compared to wtp53 cells, particularly in terms of ferroptotic cell death. However, considering that mutp53 expression was reduced by curcumin, this might suggest that such treatment could be combined with other drugs to enhance the cytotoxicity against these cells. It should be considered that, for a more efficient therapeutic effect, further studies are needed, as the bioavailability of this compound is low, mainly due to poor absorption, rapid metabolism, and systemic elimination [27].

## 4. Materials and Methods

### 4.1. Cell Cultures, Reagents, and Treatments

HCT116 and SW480, human colorectal carcinoma cell lines, were cultured in Dulbecco’s modified Eagle medium (DMEM Sigma Aldrich, St. Louis, MO, USA), supplemented with 10% fetal bovine serum (FBS; Sigma Aldrich), L-glutamine (40 mM) (Invitrogen Waltham, MA, USA), streptomycin (100 μg/mL) (Corning, New York, NY, USA), and penicillin (100 U/mL) (Corning), at 37 °C in a 5% CO_2_ incubator. Primary human colonic epithelial cells (HCoEpCs; iXCells Biotechnologies 10100 Willow Creek Rd, San Diego, CA, USA) were cultured in Epithelial Cell Growth Medium (iXCells Biotechnologies).

Cells were seeded at a density of 3 × 10^5^ cells/well in 6-well plates and, depending on the experiments, the day after were treated for an additional 24 h with: curcumin (Cur) 10, 25, and 50 μM; DS-3201 (DS, Valemetostat, Selleckchem, Cologne, Germany), an EZH2 inhibitor, 5 and 10 μM; Bix-01294 (Bix), a G9a inhibitor, 3 and 6 μM; sinefungin (Sine), a SET7/9 inhibitor, 10 and 20 μM; and MI-2, a menin inhibitor, 2.5 and 5 μM.

To inhibit phagosome–lysosome fusion or to inhibit proteasomal activity, HCT116 and SW480 cells were seeded at a density of 3 × 10^5^ cells/well in 6-well plates and, the day after, were treated with Cur 25 μM for 24 h and incubated with NH_4_Cl 40 μM or bortezomib (20 nM) during the last 6 h. In all the experiments, DMSO was added as vehicle and untreated cells were used as control (CT).

### 4.2. Cell Viability and Cell Proliferation Assays

HCT116 and SW480 cell lines were treated with Cur or with the different inhibitors as described above and cell viability was determined by trypan blue exclusion assay (Sigma-Aldrich, Burlington, MA, USA, 72571). Unstained cells (live cells) were counted by light microscopy using a Neubauer hemocytometer.

Mitochondrial activity was evaluated by an MTT cell proliferation kit (Roche, Basel Switzerland). For this assay, 4 × 10^3^ cells/well were seeded in 100 μL of complete DMEM in 96-well plates and, the day after, were treated with Cur 10, 25, and 50 μM and then cultured for an additional 24 h. Absorbance was measured by an Absorbance 96 reader (Byonoy GmbH, Hamburg, Germany). All the experiments were performed in triplicate and repeated three times.

### 4.3. Western Blotting

HCT116 and SW480 cell pellets were lysed in RIPA buffer (NaCl 150 mM, NP40 1%, Tris-HCl pH8 50 mM, deoxycholic acid 0.5%, SDS 0.1%) containing protease and phosphatase inhibitors. Subsequently, Western blotting analysis was performed as previously described [28]. Briefly, 10 µg of proteins for each sample was loaded on a pre-cast polyacrylamide gel (Bolt^TM^ 4–12% Bis-Tris Plus, Invitrogen, Waltham, MA, USA) and then transferred to a nitrocellulose membrane (Bio-Rad, Hercules, CA, USA) that was stained with red Ponceau (SERVA Electrophoresis GmbH, Heidelberg, Germany, 33,427.01). Next, in most of the experiments, membranes were cut into slices and, after 30 min in a blocking solution (PBS, Tween 20 0.1%, BSA 2%), each of them was incubated with specific primary antibodies for 1 h at room temperature or overnight at 4 °C. Membranes were then washed three times in PBS Tween 20 0.1% and subsequently incubated for 30 min with the appropriate secondary antibody conjugated to horseradish peroxidase. Finally, membranes were washed three times in PBS Tween 20 0.1% and a Western Bright ECL chemiluminescence kit (Advansta, Menio Park, CA, USA) was used for protein detection.

### 4.4. Antibodies

All primary and secondary antibodies were diluted in PBS Tween 20 0.1% containing BSA 2%. Primary antibodies used were: mouse monoclonal anti-p53 (DO-1) (1:100) (Santa Cruz Biotechnology, Dallas, TX, USA, Sc-126); mouse monoclonal anti-Ac-Lysine (AKL5C1) (1:500) (Santa Cruz Biotechnology, Dallas, TX, USA, Sc-32268); rabbit polyclonal anti-Met-Lysine (1:2.000) (Invitrogen, Waltham, MA, USA, PA5-77770); mouse monoclonal anti-β-Actin (AC-74) (1:10.000) (Sigma-Aldrich, A5441); mouse monoclonal anti-GAPDH (1: 10.000) (Santa Cruz Biotechnology, A5316); mouse monoclonal anti-SET7/9 (C-11) (1:100) (Santa Cruz Biotechnology, Sc-390823); rabbit monoclonal anti-PARP (1:500) (Cell Signalling, Danvers, MA, USA, #9542); rabbit polyclonal anti-EZH2 (1:1.000) (Proteintech, Rosemont, IL 60018, USA, #21800-1-AP); mouse monoclonal anti-G9a (C-9) (1:100) (Santa Cruz Biotechnology, Dallas, TX, USA, Sc-515726); rabbit monoclonal anti-Anti-MLL1 (1:1.000) (Bethyl Laboratories, Montgomery, TX, USA, #A300-086A). Goat anti-mouse IgG-HRP (1:20.000) (Bethyl Laboratories, #A90-116P) and goat anti-rabbit IgG-HRP (1:40.000) (Bethyl Laboratories, #A120-101P) were used as secondary antibodies.

### 4.5. Densitometric Analysis

The quantification of protein bands was performed by densitometric analysis using ImageJ software (1.47 version, NIH, Bethesda, MD, USA), which was downloaded from the NIH website (http://imagej.nih.gov, accessed on 10 February 2022).

### 4.6. Quantitation of HNE Protein Adducts as a Marker of Ferroptosis

To detect and quantify of 4-hydroxynonenal (4-HNE) protein adducts in HCT116 and SW480 cells untreated or treated with curcumin 25 μM for 24 h, the Cell Biolabs’ (San Diego, CA, USA) OxiSelect™ HNE Adduct Competitive ELISA Kit (STA-838) was used, according to the manufacturer’s instruction.

### 4.7. RNA Extraction, Reverse Transcription, and Quantitative Real-Time Polymerase Chain Reaction (qRT-PCR)

RNA was extracted from HCT116 and SW480 cells untreated or treated with Cur 25 μM or with the above-mentioned methyltransferase inhibitors for 24 h. RNA extraction was performed by using TRIzol™ Reagent (Life Technologies Corporation, Carlsbad, CA, USA), following the manufacturer’s instructions. Then, to remove DNA contamination, RNA was incubated with RNase-free DNase I 1 u/mL (Norgen Biotek Corp, Thorold, ON, Canada), for 10 min at RT. Subsequently, reverse transcription was performed by using a High-Capacity cDNA Reverse Transcription kit (Applied Biosystems, Waltham, MA, USA) and the real-time qPCR with a SensiFast SYBR No-ROX kit (Bioline, Memphis, TN, USA). In all experiments, beta-actin was used as the reference gene and the 2^−ΔΔCt^ method to normalize gene transcription data.

Primers used were:
NOXA1 Fw 5′–AATCATGGACTCCCCAAG–3′;
NOXA1 Rev 5′–GAGAGAGGAGCCTGTTTG–3′;
TP53 Fw 5′–GTGTGGAGTATTTGGATGAC–3′;
TP53 Rev 5′–GTCAGAGCCAACCTCAG–3′;
EZH2Fw 5′–CCATAAAATTCTGCTGTAGGG–3′;
EZH2 Rev 5′–AAAAATAAGAGCAGCCTGAG–3′;
G9-A Fw 5′–CAAGTCTGAAGTTGAAGCTC–3′;
G9-A Rev 5′–ATCTTCCTCTTCTTCTTCCTC–3′;
SET 7/9 FW 5′–CTGAATCTCTTATTTCCAGTGC–3′;
SET 7/9 Rev 5′–AACCTCTTGGTGTGTAATTC–3′;
MLL1 Fw 5′–AAAGACTTCTAAGGAGGCAG–3′;
MLL1 Rev 5′–AACATATAGCAACCAATGCC–3′;
ACTIN Fw 5′–TCATGAAGTGTGACGTGGACATC–3′;
ACTIN Rev 5′–CAGGAGGAGCAATGATCTTGATCT–3′.

### 4.8. Statistical Analysis

In all figures, results are shown as the mean ± standard deviation (SD) of three independent experiments. Statistical analysis was performed by Student’s *t*-test. A difference was considered statistically significant when the *p*-value was <0.05 and is indicated with * in the figures. A *p*-value ≥ 0.05 was instead considered not significant and is not indicated in the figures.

## Data Availability

The datasets generated and analyzed during the current study are available from the corresponding author upon reasonable request.

## Supplementary Materials

The following supporting information can be downloaded at: https://www.mdpi.com/article/10.3390/molecules30020335/s1, Figure S1: Cytotoxic effect of curcumin on HCT116 p53-/- and primary human colonic epithelial cells (HCoEpCs).
