# Supplementary material for: Changes in Lysine Methylation Contribute to the Cytotoxicity of Curcumin in Colon Cancer Cells
Source: Molecules. 2025 Jan 16;30(2):335. doi: 10.3390/molecules30020335 (PMC11767838; doi:10.3390/molecules30020335)
Supplement: Supplementary file 1 [file molecules-30-00335-s001.zip › molecules-3383504-supplementary.docx]

Supplementary Materials

Changes in Lysine Methylation Contribute to the Cytotoxicity of Curcumin in Colon Cancer Cells

Roberta Santarelli, Paola Currà, Michele Di Crosta, Roberta Gonnella, Maria Saveria Gilardini Montani * and Mara Cirone *

Department of Experimental Medicine, Sapienza University of Rome, 00161 Rome, Italy;
roberta.santarelli@uniroma1.it (R.S.); curra.1999148@studenti.uniroma1.it (P.C.);
michele.dicrosta@uniroma1.it (M.D.C.); roberta.gonnella@uniroma1.it (R.G.)

***** Correspondence: mariasaveria.gilardinimontani@uniroma1.it (M.S.G.M.); mara.cirone@uniroma1.it (M.C.)


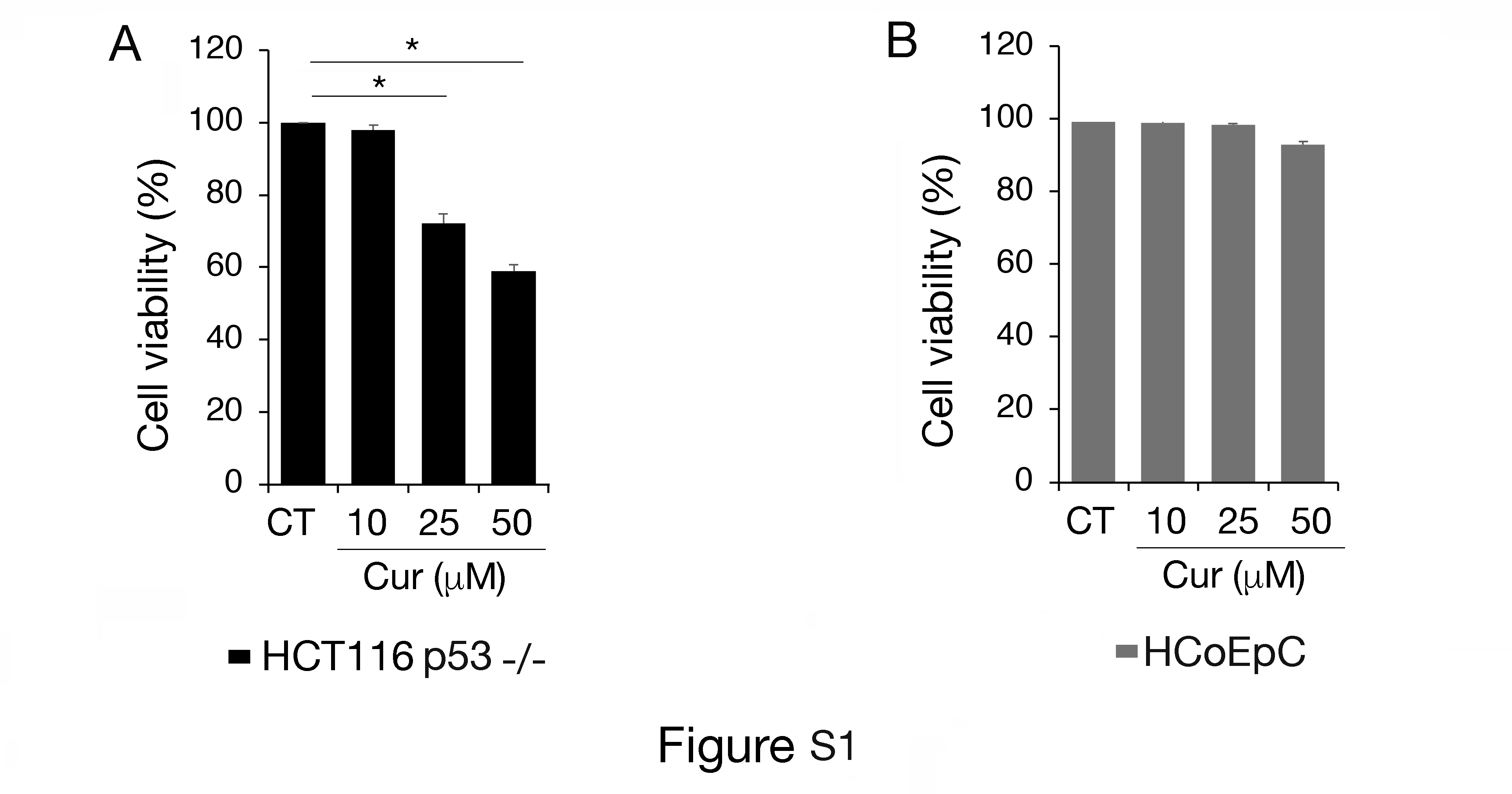


**Figure S1.** Cytotoxic effect of curcumin on HCT116 p53-/- and primary human colonic epithelial cells (HCoEpC). (A) Trypan blue assay on HCT116 p53-/- and HCoEpC cells cultured in presence of Cur 10, 25 and 50 μM for 24 hours. Untreated cells were used as control (CT). The histograms represent the mean plus SD of three different experiments. * indicates p-value <0.05.
